# Artificial Intelligence Model of Drive-Through Vaccination Simulation

**DOI:** 10.3390/ijerph18010268

**Published:** 2020-12-31

**Authors:** Ali Asgary, Svetozar Zarko Valtchev, Michael Chen, Mahdi M. Najafabadi, Jianhong Wu

**Affiliations:** 1 Disaster & Emergency Management, School of Administrative Studies, York University, Toronto, ON M3J 1P3, Canada; 2 Advanced Disaster, Emergency and Rapid Response Simulation (ADERSIM), York University, Toronto, ON M3J 1P3, Canada; mirmahdi@yorku.ca (M.M.N.); wujh@yorku.ca (J.W.); 3 Department of Mathematics and Statistics and Laboratory for Industrial and Applied Mathematics, York University, Toronto, ON M3J 1P3, Canada; zv713@mathstat.yorku.ca (S.Z.V.); chensy@mathstat.yorku.ca (M.C.)

**Keywords:** COVID-19 pandemic, artificial intelligence, drive-through, mass vaccination, discrete event simulation

## Abstract

Planning for mass vaccination against SARS-Cov-2 is ongoing in many countries considering that vaccine will be available for the general public in the near future. Rapid mass vaccination while a pandemic is ongoing requires the use of traditional and new temporary vaccination clinics. Use of drive-through has been suggested as one of the possible effective temporary mass vaccinations among other methods. In this study, we present a machine learning model that has been developed based on a big dataset derived from 125K runs of a drive-through mass vaccination simulation tool. The results show that the model is able to reasonably well predict the key outputs of the simulation tool. Therefore, the model has been turned to an online application that can help mass vaccination planners to assess the outcomes of different types of drive-through mass vaccination facilities much faster.

## 1. Introduction

Since the COVID-19 pandemic started, efforts for vaccine development and production began in many different countries. It is argued that an effective vaccine can be the best solution to end and minimize the impacts of the pandemic. While some countries have already started vaccine testing and production, it is estimated that approved vaccination solutions for large-scale implementation can be ready as soon as January 2021 or even earlier [1]. Once a vaccine becomes available, the next challenge would be the vaccination of large numbers of people in a short period of time to minimize further human and economic impacts of the pandemic [2,3]. Rapid mass vaccination requires many local vaccination clinics. Considering that a vaccine will become available for the general public use in the near future, it is important to start planning ahead of time to be able to implement mass vaccination effectively and efficiently [4]. Therefore, access to mass vaccination modeling and simulation tools has become very important for public health units that are going to plan, manage and run different types of mass vaccination facilities.

Use of drive-through vaccination has been tested during past public health emergencies such as the H1N1 and during the COVID-19 pandemic for rapid testing and it has shown some promising outcomes [5,6]. A significant advantage of the drive-through clinics is their lower virus transmission possibility compared to walk-in clinics because people are isolated in their cars and are not in direct contact with other people [7]. They are only in contact with the immunization staff that are likely vaccinated first and are equipped with personal protection equipment. However, studies show that rapid vaccination using drive-through clinics requires proper site selections and design, human resources management, and careful attention to operational and logistical details [8].

COVID-19 mass vaccination can be implemented using a combination of traditional approaches such as health clinics, pharmacies, nursing homes, schools, workplaces, places of worship, and innovative and temporary facilities such as drive-through and large stadiums, and parking lots. Use of large temporary facilities allows for rapid and safer mass vaccination [9]. Drive-through facilities have lower disease transmission, low virus exposure, have large throughput, provide better security, and are more accessible and comfortable particularly for individuals with mobility issues or are geographically dispersed [2,7,10,11,12]. The drive-through method has some limitations and shortcomings as its usage is influenced by weather conditions, requires suitable and available spaces, and significant logistical planning. Moreover, drive-through can cause traffic issues in the localities and may expose staff to carbon monoxide exposure [6]. Despite these, drive-through facilities for mass vaccinations are recommended and are being seriously considered as part of the COVID-19 immunization process [13].

Drive-through vaccination was largely used in the USA in 2009 during the H1N1 influenza pandemic. Subsequent studies found that with reasonable processing time and negligible carbon monoxide exposure, drive-through is a feasible and effective alternative to traditional walk-in clinics by providing faster vaccination under lower disease transmission risks [14,15]. Drive-through clinics have been used for testing and provision of health services such as prenatal and pharmacy services during the COVID-19 pandemic as well [16,17,18,19]. The use of drive-through during the COVID-19 pandemic demonstrated its effectiveness in the absence of enough clinical testing facilities [20].

Drive-through mass vaccination facilities can be designed and implemented in various shapes and sizes [7,19,21,22,23]. Drive-through facilities with more dispensing lanes can provide higher throughputs and prevent traffic overflow onto neighboring streets [10,24]. Drive-through facilities need to be staffed with different skills including immunization, nursing, admin, IT, logistics, and security. Drive-through clinics can be set to work 24 h a day in two, three, or four shifts. Although drive-throughs have lower disease transmission risks, especially when staff and visitors use personal protective equipment and are vaccinated before vaccinating others, attention must be paid to the safety and the security issues related to traffic, drivers’ behaviors, and extreme weather conditions. Hence, large drive-through clinics may need to be supported by local emergency services such as police, fire, and paramedics [18,25,26].

Several studies have modeled and examined mass vaccination and point of dispensing clinics using discrete event, agent-based, optimization modeling, and multi-criteria decision support systems for better layout, efficiency, resource allocation, and scheduling [2,10,22,26,27,28,29,30,31]. Some of these models have been turned into software packages and tools. Examples of such tools included Clinic Planning Model Generator developed by Aaby et al. [22], Maxi-Vac by the US Centers for Disease Control, and Real Opt by Lee et al. [10]. However, new models and tools are needed to incorporate the specific challenges posed by the COVID-19 pandemic and emerging technological solutions in simulations and artificial intelligence. This paper aims to use the advancements in simulation tools and artificial intelligence to develop an application that can help mass vaccination planners to quickly assess potential outputs of different drive-through mass vaccination clinics.

## 2. Materials and Methods 

The artificial intelligence (AI) discussed here was developed based on a big dataset derived from an existing drive-through mass vaccination simulation created by the authors [32]. In this section, we briefly explain the drive-through simulation tool and then provide some details about our machine learning model.

### 2.1. The Drive-Through Simulation

**Figure 1:**
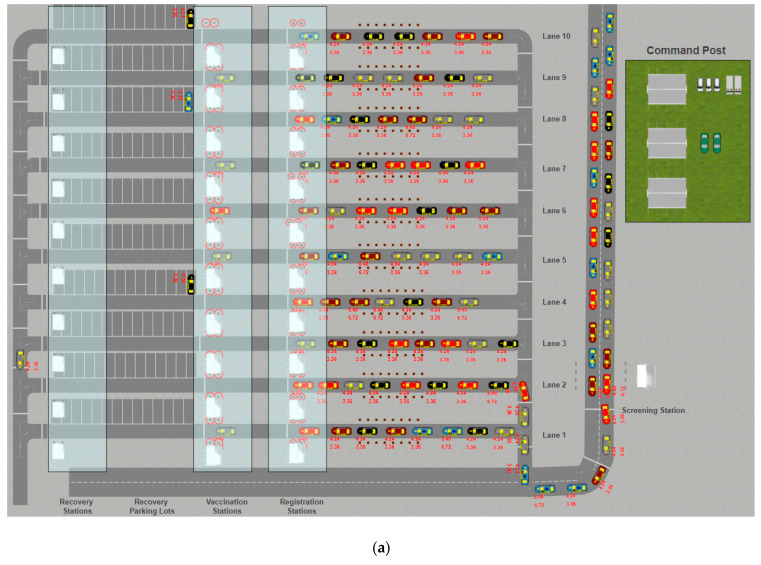

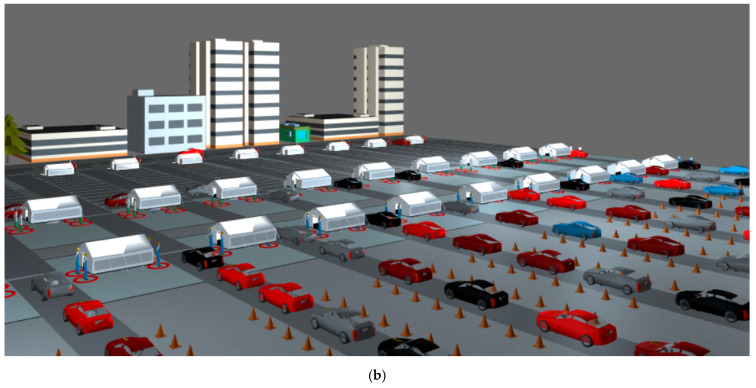
The 2D layout (**a**) and 3D (**b**) of the drive-through mass vaccination simulation tool (available at: https://cloud.anylogic.com/model/583c2075-6a8b-41be-8a03-d692eba71683).

The drive-through model used in this study is a hybrid model consisting of a discrete event and an agent-based simulation. The model contains several agents including passengers, staff, and cars. The physical layout of the drive-through (Figure 1) can be extended up to ten lanes with a maximum area of 20,000 (150 m by 130 m). Passengers entering the drive-through are screened at the screening station to ensure that they meet the vaccination criteria and receive the necessary handout materials. Cars and passengers passed the screening phase move to one of the open lanes for registration, vaccination, and recovery, which can be determined in part by the availability of High Occupancy Vehicle Lane and Pre Registered Lanes. Vaccination will be conducted by professional immunization staff at the vaccination stations. Cars move to one of the available parking spaces located after the vaccination stations. Individuals experiencing complications after vaccination can be taken care of by the relevant staff. Each station is supported by up to four kiosks.

**Figure 2:**
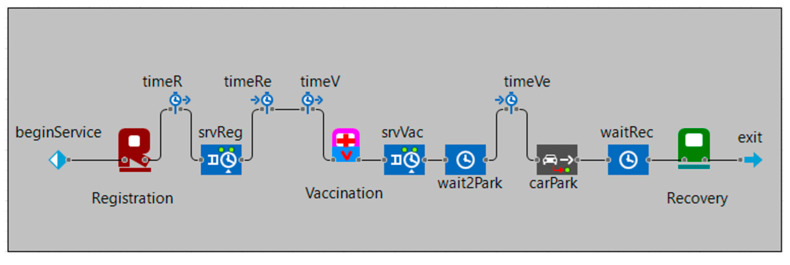
The service lanes flowchart of the drive-through mass vaccination simulation tool.

The discrete event model of the drive-through simulation generates the service process for each lane from registration to recovery (Figure 2). Start and end time in each station is measured through time blocks (for example, timer and timeRe refer to the start and end time for registration respectively).

**Figure 3:**
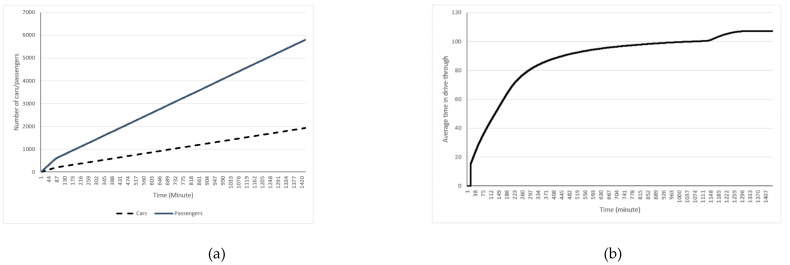
Sample simulation results for number of cars and passengers using the drive-through (**a**) and average time spent in the drive-through (**b**) based on drive-through settings in Table 1.

A sample of our drive-through simulation experiment results is presented here. Table 1 shows the parameters setting for the experiment and Figure 3 presents the output of the experiment. The values presented in Figure 3 are the average of 50 iterations of this experiment. According to these results, a total of 1933 cars with 5804 passengers will be processed in the drive-through mass vaccination during a day with three shifts of 8 h each.

### 2.2. The AI Model

The high fidelity AnyLogic model suffers at inference time, due to the computation cost of running the simulation. A single simulation run can take up to 90 s, which may not be efficient in any real time analysis tasks. Due to the stochastic nature of the drive-through simulation, a significant number of simulations runs (using Monte Carlo method) is needed for each parameter setting that requires more simulation time. In order to alleviate this, we attempt to train a neural network to predict the outputs of the simulation based on the model parameters.

As is often the case with training neural networks, the problem is rooted in gathering large amounts of data on which to train. For this, we make use of the AnyLogic parallelized computation ability, to simulate large batches of simulation runs at the same time. In doing so, we strategically sample across a large range of parameters, near the domain of interest required of real-world scenarios. Parameter simulation ranges can be found in Table 2.

We chose to sample all variables uniformly, as we do not have any distribution knowledge of the real-world application of the model. Furthermore, by sampling in such an unbiased fashion, we reduce the chances of the network overfitting based on some intrinsic property of our training set. Some conditional restrictions are built regarding the binary variables, as noted in Table 1. Altogether, we generated about 125 k simulation samples, providing us with a dense enough training domain.

## 3. Results

**Figure 4:**
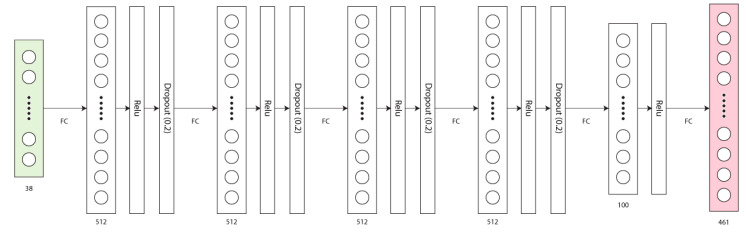
Each layer in our network model consists of a fully connected tensor with a Relu activation function. The first four all utilize a 20% dropout layer for regularization, while the final feature layer does not.

**Figure 5:**
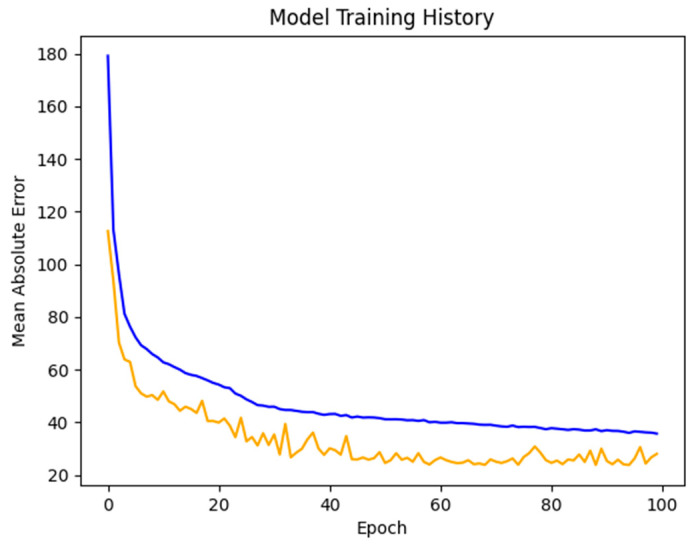
Test (blue) and validation (yellow) set errors over the course of training through 100 epochs. Validation error seems to level off around 50 epochs in.

We utilized an 80–20 train-test split to train a 5-layer feed forward neural network, which is shown in Figure 4. We trained the network using the Adam optimizer with a learning rate of 0.001, minimizing the mean absolute error, in batches of 256. Training was stopped after 100 epochs, with the test and validation error displayed in Figure 5.

**Figure 6:**
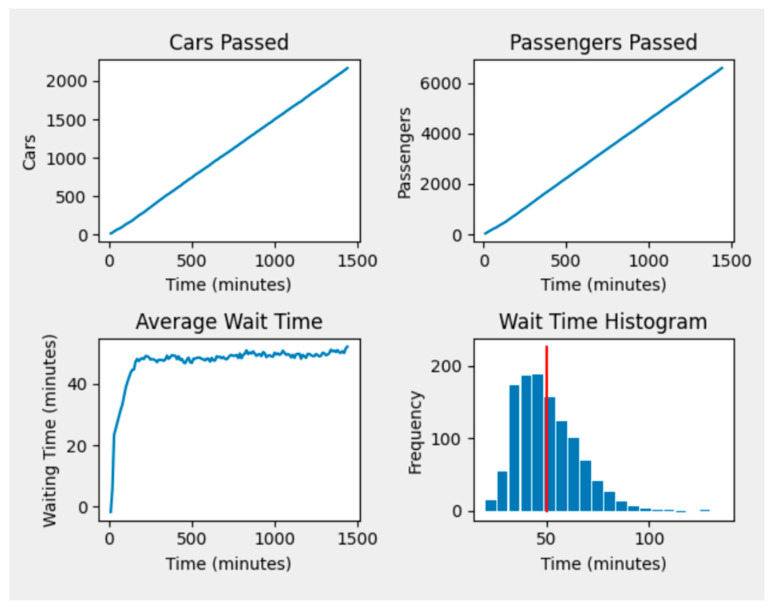
Example of our model predictions for a set of input parameters. All time data is sampled on 10 min intervals but can easily be adjusted for finer grain information. Wait time distributions align with pre-observed results directly from the AnyLogic model. Red line represents mean wait time.

Our final network is 10 Mb in size, which is small enough to be implemented even on the smallest of mobile devices and is able to infer results instantly. More precisely, by entering the parameters (within reasonable ranges) listed in Table 1, our model is able to predict information such as cars and passengers passing through the vaccination center, average wait times throughout the day and overall completion metrics. An example of this can be seen in Figure 6.

**Figure 7:**
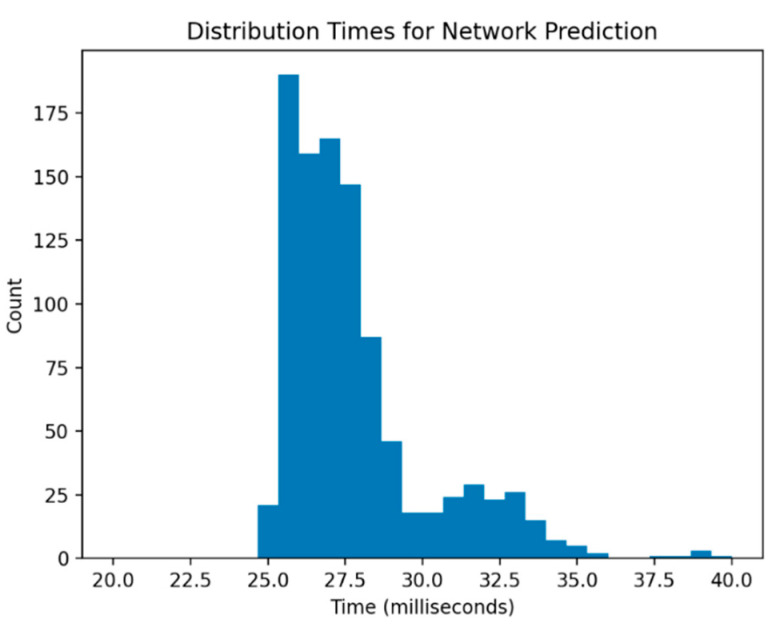
Computation times across 1000 random input samples using our fully trained neural network model.

Our neural network model is capable of prediction orders of magnitudes faster than the full simulation model. More precisely, predictions were on average computed in 0.027 s, with minimum and maximum times of 0.025 and 0.039 s, respectively, in a simulation of 1000 random input variations. Distribution of the results can be seen in Figure 7. Notice that the time scale we achieve here is on the order of milliseconds, whereas previously we required minutes to produce these same results. We achieve an improvement of over 3000× in terms of speed, largely due to the computation cost of simulating the entire event. Furthermore, we do all this locally as opposed to the need for cloud computation.

## 4. Discussion 

The drive-through model developed in this study is a meta-model (or models of models) that as described by Obsie et al. [33], represents a deterministic proxy for stochastic simulation models. However, developing this type of meta-models requires running complex simulation for many combinations and then using the data generated by each run to create a simpler machine learning model that is a reasonable approximation of the initial simulation model. The meta-models can then provide quick predictions for different input values. While simulation-based or meta-modeling is not a new concept [34], its application in different fields and simulation types are still limited. In this study, we applied meta-modeling approach in the context of mass vaccination.

The AI-based drive-through model developed here has been deployed as a web-application that can be used by potential users (www.adersim.org). The approach serves our purpose of providing a general decision supporting tool for planning a large-scale drive-through vaccination. A general decision unit, such as a municipal government or a public health region, may simply input its unique parameters, and our model will generate accurate prediction of waiting time, capacity, etc., very quickly. Since our AI model is much faster than the simulation model, municipal governments or public health regions may compare multiple decisions, regarding the number of lanes, staffing levels, etc., and make the best choice. Further, our AI model is as small as 10 Mb, which can be implemented on any mobile device as well.

Our drive-through simulation has been developed and parameterized based on the past and current best practices available for drive-through design, settings, and operations. However, because the details of a potential SARS-Cov-2 vaccination protocols are not available yet, our drive-through mass vaccination simulation tool can be further tuned as new information becomes available. For example, the vaccination and recovery times may be very much influenced by the specific immunization protocols for SARS-Cov-2 vaccine, which may be different from previous vaccination. In the absence of real drive-through cases, such simulations can play a significant role in pre-planning.

Another potential application of our method in the mass immunization case would be an extension of this approach for other mass vaccination clinics such as large temporary walk-in clinics that is currently under development by the authors. In future, we would like to further refine our neural network to further reduce its complexity and enhance its accuracy. More importantly, it will be interesting to see whether the same network will serve more general cases as they arise in future.

## 5. Conclusions

Drive-through clinics may be able to play a significant role in SARS-Cov-2 mass vaccination process as they provide a faster and safer options, as proven to be effective for SARS-Cov-2 testing in different countries. As such, drive-through option can complement other traditional and new mass immunization methods.

In this paper, we demonstrated how data generated from a simulation can be used to develop a new and better predictive machine learning model for drive-through mass vaccination clinics. The generated model can be used to obtain quick predictions of the number of people to be vaccinated and the average time it takes for vaccination under various parameter settings. The results show promising outcomes in applying this method in other aspects of pandemic management where large numbers of simulations are developed for prediction. Most of these simulations have the potential to be further enhanced and turned into artificial intelligence models that can help end users and policy makers to assess the impacts of various policy options.

## Figures and Tables

**Table 1:** Parameter settings for the sample drive-through simulation experiment.

Parameters	Value
Lane 1-Lane10	Open
Minimum number of passengers in cars	1
Maximum number of passengers in cars	5
Number of cars coming to drive through per min	5
Average registration time (min)	4.24
Average vaccination time (min)	3.26
Average recovery time (min)	4
Number of staff in each station in lane 1-lane10	4
Assign High Occupancy Lanes (HOV)	FALSE
Fraction of cars pre-registered for vaccination (0–1)	50
Pre-registration impact factor (0–1)	0.5
Consider pre-registration	TRUE
Low occupancy vehicle	FALSE
Fraction of non-adult passengers	0.15
Fraction of cars rejected at screening	0.01
Shift hours	8
Number of shifts	3
Average screening time	0.5
Minimum screening time	0.25
Maximum screening time	0.5
Dynamically learn and adjust cars going to each lane	TRUE
Use schedule for incoming cars	FALSE

**Table 2:** Parameter sampling ranges for model training.

Parameter	Range	Notes
Average recovery time (min)	5–10	
Average registration time (min)	2–7	
Average screening time	0.25–1	
Maximum screening time	1	fixed
Minimum screening time	0.25	fixed
Average vaccination time (min)	2–7	
Maximum number of passengers in cars	5–7	integer only
Minimum number of passengers in cars	1	fixed
Fraction of non-adult passengers	0.15–0.30	
Number of cars coming to drive through per minute	0.5–5	
Fraction of cars rejected at screening	0.01	fixed
Shift hours	6, 8 or 12	
Number of shifts	2, 3 or 4	Depending on shift hours such that total hours = 24
Days	1	fixed
Lanes 1–10	True or False	At least 1 lane is always open
Number of staff in each lane	1–4	Can differ lane to lane, integer only
Consider pre-registration	True or False	
Assign High Occupancy Lanes (HOV)	True or False	
Dynamically learn and adjust cars going to each lane	True or False	Can only be true if HOV is open
Low occupancy vehicle	True or False	Can only be true if HOV is open

## Data Availability

The data presented in this study are available on request from the corresponding author. The data are not publicly available due to its huge size.
